# Blood Oxygenation Level-Dependent CMR-Derived Measures in Critical Limb Ischemia and Changes With Revascularization

**DOI:** 10.1016/j.jacc.2015.10.085

**Published:** 2016-02-02

**Authors:** Adnan Bajwa, Roman Wesolowski, Ashish Patel, Prakash Saha, Francesca Ludwinski, Mohammed Ikram, Mostafa Albayati, Alberto Smith, Eike Nagel, Bijan Modarai

**Affiliations:** a Academic Department of Vascular Surgery, Cardiovascular Division, King’s College London, BHF Centre of Research Excellence & NIHR Biomedical Research Centre at King’s Health Partners, St. Thomas’ Hospital, London, United Kingdom; b Department of Cardiovascular Imaging, Division of Imaging Sciences and Biomedical Engineering, King’s College London, BHF Centre of Research Excellence, Wellcome Trust−EPSRC Medical Engineering Centre & NIHR Biomedical Research Centre at King’s Health Partners, St. Thomas’ Hospital, London, United Kingdom; c Institute for Experimental and Translational Cardiovascular Imaging, DZHK Centre for Cardiovascular Imaging, University Hospital Frankfurt, Goethe University Frankfurt, Frankfurt am Main, Germany

**Keywords:** angioplasty, cardiovascular magnetic resonance, perfusion, surgery, ABPI, ankle-brachial pressure index, ASL, arterial spin labeling, BOLD, blood oxygenation level-dependent, C:F, capillary-fiber ratio, CLI, critical limb ischemia, CMR, cardiovascular magnetic resonance, DCE, dynamic contrast enhanced, Grad, gradient during reactive hyperemia, GRE, gradient recalled echo, PAD, peripheral arterial disease, SRi, signal reduction during ischemia

## Abstract

**Background:**

Use of blood oxygenation level-dependent cardiovascular magnetic resonance (BOLD-CMR) to assess perfusion in the lower limb has been hampered by poor reproducibility and a failure to reliably detect post-revascularization improvements in patients with critical limb ischemia (CLI).

**Objectives:**

This study sought to develop BOLD-CMR as an objective, reliable clinical tool for measuring calf muscle perfusion in patients with CLI.

**Methods:**

The calf was imaged at 3-T in young healthy control subjects (n = 12), age-matched control subjects (n = 10), and patients with CLI (n = 34). Signal intensity time curves were generated for each muscle group and curve parameters, including signal reduction during ischemia (SRi) and gradient during reactive hyperemia (Grad). BOLD-CMR was used to assess changes in perfusion following revascularization in 12 CLI patients. Muscle biopsies (n = 28), obtained at the level of BOLD-CMR measurement and from healthy proximal muscle of patients undergoing lower limb amputation (n = 3), were analyzed for capillary-fiber ratio.

**Results:**

There was good interuser and interscan reproducibility for Grad and SRi (all p < 0.0001). The ischemic limb had lower Grad and SRi compared with the contralateral asymptomatic limb, age-matched control subjects, and young control subjects (p < 0.001 for all comparisons). Successful revascularization resulted in improvement in Grad (p < 0.0001) and SRi (p < 0.0005). There was a significant correlation between capillary-fiber ratio (p < 0.01) in muscle biopsies from amputated limbs and Grad measured pre-operatively at the corresponding level.

**Conclusions:**

BOLD-CMR showed promise as a reliable tool for assessing perfusion in the lower limb musculature and merits further investigation in a clinical trial.

Peripheral arterial disease (PAD) affects 27 million people in North America and Europe (1) and is characterized by progressive development of arterial stenoses and occlusions. Most individuals with PAD are asymptomatic (2), with the remainder developing intermittent claudication or critical limb ischemia (CLI). The latter is characterized by pain at rest, ulceration or gangrene, and high morbidity and mortality. Up to 25% of patients eventually lose a limb (3). Patients with PAD, irrespective of symptoms, have an increased risk of myocardial infarction and stroke and are 6× more likely to die within 10 years (4).

The main treatments for patients with CLI and intermittent claudication are bypass surgery or angioplasty/stenting, which relieve symptoms and promote limb salvage. Imaging methods, such as duplex ultrasonography, cardiovascular magnetic resonance (CMR), computed tomography (CT), and intra-arterial angiography inform the extent of disease, aid in planning interventions, and permit assessment of patency post-revascularization. These imaging modalities show the severity of luminal disease in the major peripheral blood vessels, but do not provide information on the microcirculation or degree of muscle perfusion in the affected limb (5), which is the most important determinant of limb salvage in patients with CLI.

Currently, no reliable method for measuring the adequacy of lower-limb perfusion exists. A surrogate for perfusion in the limb, ankle-brachial pressure index (ABPI) only crudely indicates flow in major leg vessels and is of limited value in the presence of calcified arteries. An objective, noninvasive method of measuring and mapping muscle perfusion would aid diagnosis and treatment of PAD and may improve limb salvage rates. This has stimulated studies using CMR for segmental, serial assessment of perfusion in the lower limb musculature 6, 7, 8, 9, 10, 11, 12, 13, 14.

Blood oxygenation level-dependent (BOLD), arterial spin labeling (ASL), and dynamic contrast enhanced (DCE) are different CMR techniques that can measure segmental perfusion in the lower limb. BOLD uses the paramagnetic properties of deoxygenated hemoglobin that induces inhomogeneities in the local magnetic field, resulting in reduced T2* relaxation times. This was first demonstrated when mapping areas of brain activation, where blood oxygenation is inversely proportional to T2* relaxation time 15, 16. BOLD-CMR has subsequently been used to delineate areas of poorly perfused myocardium 17, 18, but the lack of reproducibility and objectivity when measuring perfusion in the lower limb has limited its utility (12).

The present study aimed to: 1) test the feasibility and reproducibility of using BOLD-CMR to measure perfusion in the lower limb; 2) use BOLD-CMR to compare perfusion in the critically ischemic and contralateral (asymptomatic) limb in the same patient, as well as in limbs of age-matched and young healthy control subjects; 3) compare changes in perfusion, measured with BOLD-CMR, before and after successful revascularization in patients with CLI; and 4) to provide preliminary histological validation of BOLD-CMR–derived parameters.

## Methods

This study complies with the Declaration of Helsinki and has ethical approval for the recruitment of patients and volunteers (Guy’s & St. Thomas’ NHS Foundation Trust−10/H0804/67). All subjects were provided with written information regarding the study, and written consent was obtained.

Patients with a clinical diagnosis of CLI (confirmed by CT angiography or duplex ultrasonography) were recruited. Age-matched subjects with no clinical evidence of PAD and young, healthy volunteers <30 years of age were used as control subjects.

Exclusion criteria included: 1) presence of cardiac pacemaker/defibrillator or other magnetically active implants; 2) severe claustrophobia; 3) fixed flexion deformities of the lower limb where the subject is unable to place the leg flat on the CMR table; and 4) patients unable to consent.

### Imaging protocol

**Figure 1:**
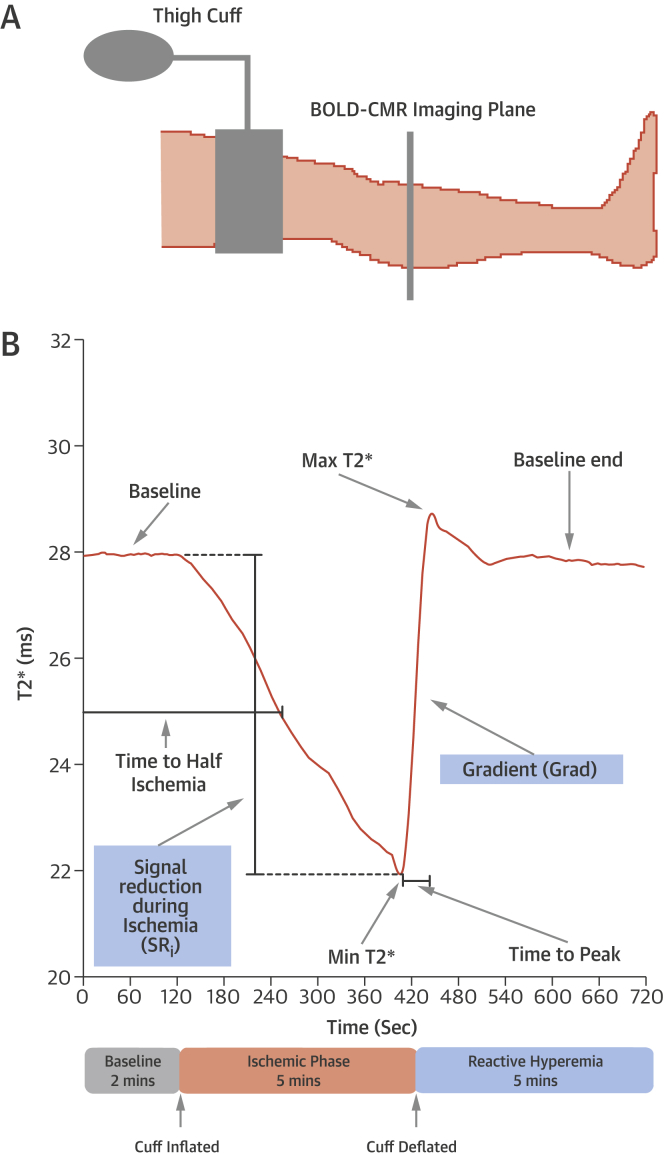
Protocol for Evoking Reactive Hyperemia **(A)** Subjects were imaged supine with an occlusive cuff placed around both thighs to provoke reactive hyperemia. The axial image slice is acquired at the widest part of the lower leg. **(B)** In this typical signal intensity time course curve from a healthy volunteer, a steady T2* signal occurs during the baseline phase (2 min) and declines in intensity during the ischemic phase (5 min). After cuff deflation, a reactive hyperemic response is seen with an increase in T2* and overshoot before returning to baseline (5 min). Curve parameters analyzed include gradient (Grad), measured by taking the mean of the highest 10 values of the first derivative during reactive hyperemia, and signal reduction during ischemia (SRi), measured as a percentage reduction in T2* signal from a mean of the baseline to a minimum T2* at the end of the ischemic phase. BOLD = blood oxygenation level-dependent; CMR = cardiovascular magnetic resonance.

To standardize scans, all subjects refrained from caffeine and exercise 4 h before imaging and rested supine for 5 min. All subjects were imaged with a 3-T Philips Achieva scanner (Philips Healthcare, Best, the Netherlands) using a multi-echo single-shot gradient recalled echo (GRE) sequence. Reactive hyperemia, instigated by cuff-induced arterial occlusion followed by rapid cuff deflation, was used to elicit T2* changes (Figure 1A, Online Appendix). A 10-mm slice at the widest part of the calf was acquired, with a field of view including both legs. Parameters for imaging were as follows: time to repeat 66 ms, 14 echoes with time to echo 4.6 ms, change in echo time 4.6 ms, flip angle 20°, field of view 288 × 288, and voxel size 1.1 × 1.1 × 10 mm^3^. Image acquisition for the 14 echoes was every 3 s. T2* maps were reconstructed by a Maximum Likelihood Estimate fit with Rician noise correction, where noise is read in k-space, for precise and unbiased automated estimation of T2*. A T2-weighted turbo spin echo (TSE) image was also acquired of the same region of the calf to allow for accurate delineation of muscle compartments.

Regions of interest (ROIs) were drawn around the 5 muscle groups (anterior, lateral, soleus, gastrocnemius, and deep posterior) in each leg, using the GRE-BOLD T2* maps with the TSE images providing a visual guide. All ROIs were adjusted for any motion artifact and volume changes in the limb. Bespoke MATLAB (MathWorks, Natick, Massachusetts) routines were used to automatically generate time-course curves for individual ROIs and analyze curve parameters. In patients with CLI, both the ischemic limb and the contralateral, asymptomatic limb were analyzed. The parameters assessed are shown in Figure 1B and the Online Appendix. T2* values were normalized to baseline values obtained prior to cuffing to allow comparisons between subjects.

### BOLD-CMR performance and analysis

Independent analysis of BOLD-CMR images by 2 blinded users was used to assess interuser variability of scans from 7 healthy volunteers, 5 of whom had repeat scans carried out 1 to 5 weeks apart to assess interscan variability. The tibial tuberosity was used as an anatomic reference point to ensure imaging at the same calf level. These images were analyzed by the same blinded user.

BOLD-CMR was performed before and 1 to 14 days (median 3 days) after revascularization in 13 patients with CLI. Intervention involved endovascular and/or open surgery of the aortic, iliac, common femoral, or superficial femoral arteries (SFAs). Patients undergoing distal bypass were excluded because of possible risk of graft occlusion with cuffing and because hematoma secondary to open surgical intervention below the knee made interpretation of BOLD-CMR scans difficult. Successful revascularization of the target vessels was confirmed by duplex and/or CT angiography and related to ABPI measured before and after intervention.

BOLD-CMR curve parameters were compared between patients who had a patent SFA distal to the recanalized inflow vessels and those in whom the SFA remained occluded to assess their sensitivity to incremental changes in blood flow.

Muscle biopsies from 3 CLI patients undergoing major lower limb amputation were taken from healthy muscle proximal to the level of amputation (n = 13) and from the corresponding level of BOLD-CMR imaging (n = 15). Used as an anatomic landmark, tibial tuberosity helped identify the level of imaging with BOLD-CMR and biopsies taken from this level. The capillary-fiber (C:F) ratio was assessed by CD31 and laminin staining. The C:F ratios obtained at the level of BOLD-CMR imaging were normalized to the C:F ratio in the healthy proximal muscle in each patient and correlated with corresponding gradient during reactive hyperemia (Grad) and signal reduction during ischemia (SRi) values (Online Appendix).

### Statistical analysis

Statistical analysis was carried out using SPSS version 20 (IBM Corp., Armonk, New York) and Prism 5 (GraphPad Software Inc., La Jolla, California). A 1-way analysis of variance (with Tukey post-hoc test) was used to analyze curve parameters. Reproducibility was assessed using intraclass correlation coefficient (ICC), where an ICC >0.9 was deemed as excellent level of agreement, and Bland-Altman analysis. Reproducibility and analysis of pre- and post-intervention changes in curve parameters was assessed by paired Student *t* test. C:F ratio comparisons between well and poorly perfused muscle was analyzed using unpaired Student *t* test. Correlation analysis of curve parameters against C:F ratio and ABPI was performed using Spearman’s correlation. Curve parameters were compared in patients with a patent or occluded SFA using a Mann Whitney *U* test. All values are given as mean ± SD, and p < 0.05 was deemed statistically significant.

## Results

A total of 37 patients with CLI, 10 age-matched control subjects, and 12 young healthy control subjects were recruited into the study (Table 1, Online Figure 1). Three CLI patients were excluded, as they were unable to lie supine long enough to undergo CMR because of rest pain severity.

**Table 1:** Demographics

	Young Control Subjects (n = 12)	Age-Matched Control Subjects (n = 10)	CLI Patients (n = 37)
Age, yrs	25 (24–28)	67 (52–71)	66 (37–86)
Male to female ratio	8:4	7:3	29:8
Smoker	1	1	26
Hypertension	0	2	29
Hypercholesterolemia	0	1	20
Diabetes	0	0	13
Rutherford classification			
IV			22
V			9
VI			6

Values are median (range) or n.

CLI = critical limb ischemia.

**Figure 2:**
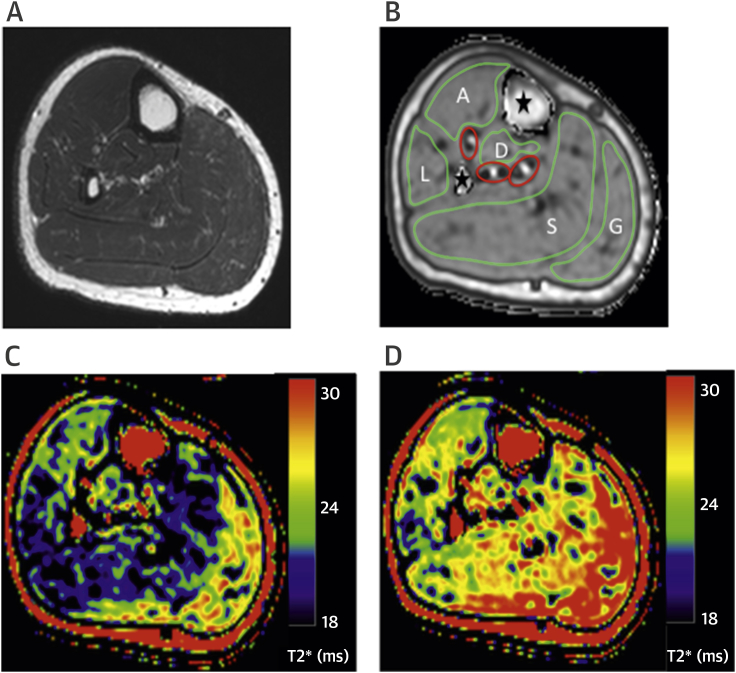
BOLD T2* Mapping CMR and Perfusion Maps **(A)** T2-weighted anatomic images allow for more accurate delineation of muscle compartments when selecting regions of interest. **(B)** Gradient recalled echo (GRE) sequence for accurate mapping of T2* signal affords high spatial resolution allowing for accurate delineation on the T2* maps of different muscle groups in the lower leg (**green outlines**: A = anterior; L = lateral; S = soleus; G = gastrocnemius; D = deep) and exclusion of major blood vessels **(red outline)**, bone **(star)**, and other soft tissue. T2* perfusion maps demonstrate changes in perfusion as measured by T2* signal intensity in the calf of a chronic limb ischemia (CLI) patient during cuffing **(C)** and reactive hyperemia **(D)**. Increased signal intensity is seen in all muscle groups after release of the cuff. Abbreviations as in Figure 1.

A T2-weighted TSE image allowed accurate visual delineation of muscle groups (Figure 2A). A high spatial resolution was achieved with the GRE-BOLD-CMR sequence, which allowed for accurate exclusion of major blood vessels, bone, and soft tissue from muscle ROIs (Figure 2B). T2* perfusion maps highlight changes in muscle perfusion during cuffing and reactive hyperemia (Figures 2C and 2D).

Automated analysis identified Grad and SRi as the most informative in discriminating between ischemic and patient contralateral limbs (p < 0.0001 for both) (Table 2, Online Appendix).

**Table 2:** Analysis of Curve Parameters

	Patient Contralateral Limbs	Ischemic Limbs	p Value
Grad, ms/s	0.28 ± 0.14	0.17 ± 0.11	0.0001
SRi, %	11.03 ± 5.62	8.68 ± 5.11	0.0001
Minimum T2*, ms	21.99 ± 3.63	22.80 ± 3.62	0.01
Baseline (start), ms	24.70 ± 4.09	25.04 ± 3.92	0.16
Baseline (end), ms	24.64 ± 4.07	24.85 ± 3.93	0.43
Maximum T2*, ms	24.93 ± 4.25	24.56 ± 3.93	0.20
TTP, s	68.7 ± 22.76	64.5 ± 29.7	0.14
THIM, s	205.5 ± 78.3	195.5 ± 81.5	0.24

Values are mean ± SD.

Grad = gradient during reactive hyperemia SRi = signal reduction during ischemia; THIM = time to half ischemia; TTP = time to peak.

### BOLD-CMR reproducibility

**Figure 3:**
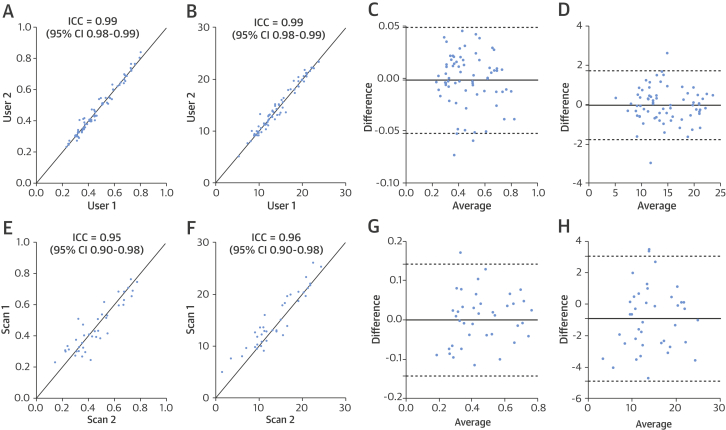
Reproducibility of BOLD-CMR Scans **(A to D)** Interuser reproducibility was tested using intraclass correlation coefficient (ICC) by 2 different users analyzing identical scans from 7 healthy volunteers and Bland-Altman analysis. **(E to H)** Interscan reproducibility was determined by analysis of interval scans that were carried out 1 to 5 weeks apart by a single user in 5 subjects. Bland-Altman analysis confirmed good reproducibility for Grad **(C for A, G for E)** and SRi **(D for C, H for F)**. CI = confidence interval; other abbreviations as in Figure 1.

There was good interuser (A.B., R.W.) reproducibility as evidenced by strong correlations for both Grad and SRi measurements (ICC: 0.99; 95% confidence interval [CI]: 0.98 to 0.99; and ICC: 0.99; 95% CI: 0.98 to 0.99, respectively; p < 0.0001 for both) (Figures 3A and 3B). Bland-Altman analysis concurred with ICC (Grad: mean bias −0.002; 95% limits of agreement: −0.05 to 0.05; SRi: mean bias −0.04; 95% limits of agreement: −1.79 to 1.70) (Figures 3C and 3D). Similar T2* curves were generated by the 2 users. No significant difference was found between Grad and SRi measured by each of the 2 investigators (Online Figure 2A).

There also were excellent interscan (A.B.) correlations for Grad and SRi in the same subject (ICC: 0.95; 95% CI: 0.90 to 0.98; and ICC: 0.96; 95% CI: 0.90 to 0.98, respectively; p < 0.0001 for both) (Figures 3E and 3F). Bland-Altman analysis confirmed good reproducibility between scans for Grad (mean bias: 0.0004; 95% limits of agreement: −0.14 to 0.14) and SRi (mean bias: −0.92; 95% limits of agreement: −4.88 to 3.04) (Figures 3G and 3H). No significant difference was found for Grad and SRi between interval scans for all muscle groups analyzed (Online Figure 2B).

**Figure 4:**
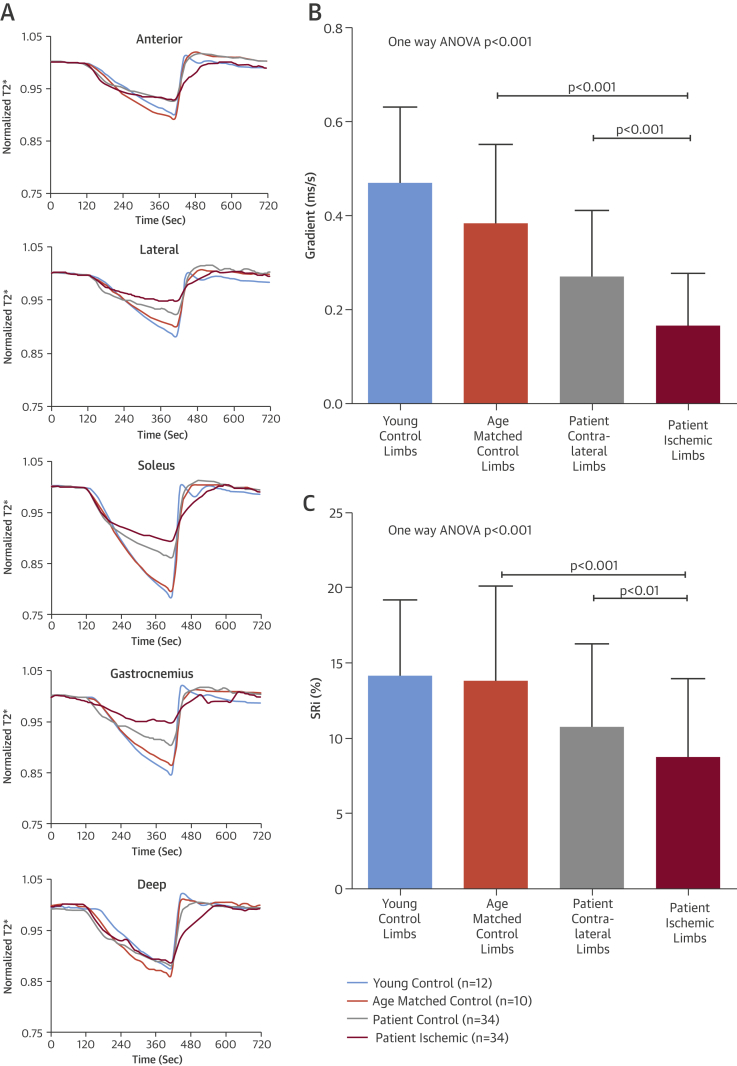
BOLD-CMR T2* Signal Changes **(A)** Mean signal intensity time course curves for the 5 muscle groups (anterior, lateral, soleus, gastrocnemius, and deep) highlight differences in T2* signal intensity provoked by reactive hyperemia. Consistent differences, particularly during ischemia and reactive hyperemia, are seen among the young control subjects **(blue)**, age-matched control subjects **(orange)**, the patient’s contralateral limb **(gray)**, and the patient’s ischemic limb **(red)**. Values (mean ± SD) for Grad **(B)** and SRi **(C)** are significantly lower in the ischemic limb compared with the patients’ contralateral limb, age-matched control subjects, and young healthy control subjects. ANOVA = analysis of variance; other abbreviations as in Figure 1.

Comparison of the T2* signal intensity curves for each of the 5 muscle groups demonstrated a clear difference between ischemic limbs and control limbs, with the soleus muscle generating the most distinctive curve characteristics (Figure 4A). In all groups, T2* signal fell after cuff inflation during the ischemic phase, but the signal for the ischemic limbs plateaued sooner than the other groups and the total reduction in signal intensity was much less. During reactive hyperemia, the rise in T2* signal was faster in the young control subjects and most delayed in the ischemic limbs of CLI patients. A significantly lower Grad was measured in the ischemic limb (0.17 ± 0.11 ms/s) compared with the patient contralateral (0.28 ± 0.14 ms/s; p < 0.001), age-matched control subjects (0.38 ± 0.17 ms/s; p < 0.001), and young control limbs (0.47 ± 0.16 ms/s; p < 0.001) (Figure 4B). Similarly, the SRi was lower in the ischemic limb (8.68 ± 5.11%) compared with the patient contralateral (11.03 ± 5.62%; p < 0.001), age-matched control subjects (13.77 ± 6.33%; p < 0.001), and young control subjects (14.07 ± 5.09%; p < 0.001) (Figure 4C).

The ABPI, measured in the ischemic limb of CLI patients, was correlated with corresponding Grad and SRi values. ABPI was only measurable in 19 of the 34 CLI patients in whom BOLD-CMR was performed. Either calcification in the below-knee arteries or absence of a Doppler signal precluded reliable measurement in the remaining 15 patients. The measured ABPI values did not correlate with either Grad or SRi (Grad: r = 0.34 and SRi: r = 0.07; p > 0.05 for both) (Online Figures 3A and 3B).

Differences in T2* response and curve parameters was analyzed in CLI patients for age, smoking history, and diabetes. CLI patients age <65 years had significantly higher Grad and SRi compared with patients age ≥65 years (p < 0.005 for both). No significant difference in Grad and SRi was seen between smokers and nonsmokers or patients with or without diabetes (Online Figure 4).

### BOLD-CMR measurement of limb perfusion after revascularization

**Figure 5:**
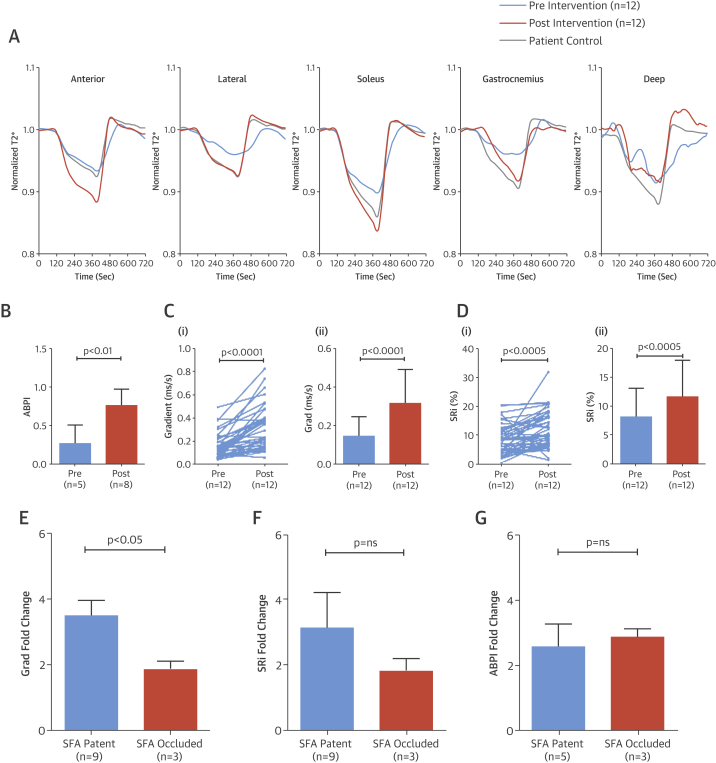
T2* Signal Changes After Limb Revascularization **(A)** Mean T2* signal intensity time course curves, shown for the 5 muscle groups in CLI patients’ ischemic limb (n = 12) pre- **(blue)** and post-revascularization **(orange)**, improve significantly after intervention, with curves from revascularized limbs changing to resemble control limbs **(gray)**. Revascularization also significantly improved ankle-brachial pressure index (ABPI) **(B)**, Grad **(C),** SRi **(D)** (ii = mean ± SD), and Grad-fold change in patients with a patent versus occluded superficial femoral artery (SFA) **(E)**. No significant changes were seen in SRi-fold **(F)** or ABPI-fold **(G)** change when comparing patent and occluded SFAs. Abbreviations as in Figures 1 and 2.

BOLD-CMR was carried out prior to and after revascularization in 13 patients (Online Table 1). One patient was excluded because of the presence of significant edema on both pre- and post-intervention imaging. T2* curves in the ischemic limb generated prior to revascularization were consistent with curves typical of limbs with impaired perfusion (Figure 5A). This was confirmed by significantly lower Grad and SRi measurements compared with both the contralateral asymptomatic legs (Grad: 0.14 ± 0.10 ms/s vs. 0.28 ± 0.14 ms/s; p < 0.0001; and SRi: 8.08 ± 5.04% vs. 11.03 ± 5.62%; p < 0.005) and age-matched control subjects (Grad: 0.38 ± 0.17 ms/s; p < 0.0001; and SRi: 13.77 ± 6.33%; p < 0.0001).

Revascularization resolved rest pain in all patients and successfully healed tissue loss in all but 1 patient, who required bypass surgery because of stent occlusion 3 months post-intervention. Improved ABPIs were measured in 8 patients. In 5 patients, ABPI was not measurable either pre- or post-operatively due to crural vessel calcification (Figure 5B).

Repeat BOLD-CMR after revascularization resulted in T2* curves consistent with improved perfusion, resembling those generated from the patient contralateral limbs (Figure 5A). There were significant improvements in Grad and SRi after limb revascularization (Grad: 0.14 ± 0.10 ms/s vs. 0.32 ± 0.18 ms/s; p < 0.0001; and SRi 8.08 ± 5.04% vs. 11.57 ± 6.31%; p < 0.005) (Figures 5C and 5D), suggesting that intervention had increased perfusion in the calf.

Patients in whom the SFA was patent (n = 9) demonstrated a larger-fold increase in Grad compared with those in whom the SFA remained occluded (n = 3) after proximal revascularization (3.49 ± 2.56 vs. 1.87 ± 0.73; p < 0.05) (Figure 5E). A similar trend was observed for SRi (3.12 ± 6.16 with patent SFA vs. 1.81 ± 1.27 with occluded SFA; p > 0.05) (Figure 5F). Patency of the SFA had no significant effect on the fold change in ABPI after intervention (2.56 ± 1.61 with patent SFA vs. 2.86 ± 0.49 with occluded SFA; p > 0.05) (Figure 5G). The change in ABPI that occurred after limb revascularization did not correlate with changes in either Grad or SRi. (r = 0.05 and r = 0.07, respectively; p > 0.05 for both) (Online Figures 5A and 5B).

**Figure 6:**
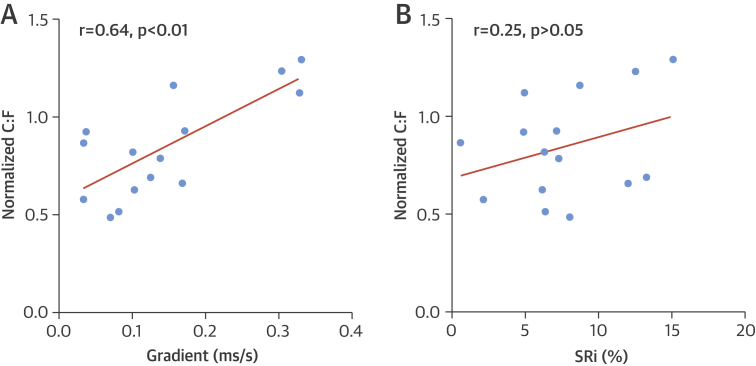
Histological Validation of BOLD-CMR–Derived Parameters The capillary-fiber (C:F) ratio in the poorly-perfused calf musculature of amputated limbs correlates with corresponding Grad **(A)** measurements but not with SRi **(B)**. Abbreviations as in Figure 1.

Analysis of biopsies from well-perfused muscle proximal to the amputation level shows a significantly higher C:F ratio compared with biopsies from the level of BOLD-CMR imaging (2.97 ± 0.79 vs. 4.15 ± 0.92; p < 0.001) (Online Appendix, Online Figure 6). The Grad and SRi values measured at calf level in the amputated limbs were correlated with C:F ratios at the corresponding level. There was a significant correlation between Grad and C:F ratio (r = 0.64; p < 0.01), but none was observed with SRi (r = 0.25; p > 0.05) (Figure 6).

## Discussion

We believe that the present study is the first to consistently demonstrate that BOLD-CMR is an objective and reproducible tool for measuring muscle perfusion in the lower limb. Distinct T2* curve parameters were consistently obtained from critically ischemic limbs and control subjects, allowing typical values to be assigned to each group. In the largest cohort of patients studied to date, BOLD-CMR showed an improvement in perfusion after successful limb revascularization (Central Illustration).

**Central Illustration:**
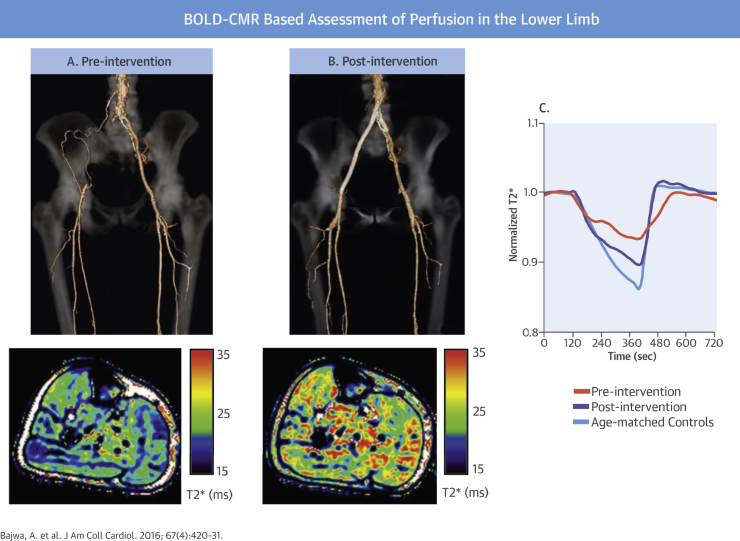
Critical Limb Ischemia and Changes With Revascularization **(A)** Computed tomographic angiogram demonstrates right common and external iliac artery occlusion **(red dashed line)** in a patient with critical limb ischemia. T2* signal heat map during reactive hyperemia shows low perfusion in all muscle groups in the right calf. **(B)** Blood oxygenation level-dependent cardiovascular magnetic resonance (BOLD-CMR), performed after right femoral endarterectomy and iliac angioplasty/stenting, detects improved perfusion in all calf muscle groups. **(C)** Typical T2* signal intensity curves are shown prior to and after limb revascularization.

BOLD-CMR is superior to ASL, an alternative noncontrast CMR-based technique, for measuring perfusion because it has a better signal-to-noise ratio and requires a shorter scan time, reducing motion artifact (5). Unlike DCE-CMR, BOLD does not require the administration of gadolinium-based contrast agents, avoiding the possibility of nephrogenic systemic fibrosis in patients with renal impairment. One advantage of ASL and DCE sequences is that, unlike BOLD, they allow absolute quantification of perfusion, but this is dependent on tracer kinetic modeling, which can introduce inaccuracies (19).

To determine whether BOLD-CMR–derived parameters (Grad and SRi) reflect the extent of tissue vascularity, these were correlated with the C:F ratio determined using histological analysis of corresponding muscle biopsies from amputated limbs. Only Grad showed a positive correlation with C:F ratio, suggesting that this parameter may be sensitive to the extent of tissue vascularity in the ischemic calf.

The GRE-BOLD-CMR sequence-based strategy used in the present study is superior to standard BOLD sequences because: 1) it achieves a higher spatial resolution, allowing for accurate exclusion of major blood vessels, bone, and other soft tissue, which avoids erroneous T2* quantification caused by inadvertently including these structures; 2) the larger number of echoes and imaging at 3-T enables more accurate quantification of T2* (20); and 3) it uses semiautomated analysis of signal intensity curves that improves reproducibility and objectivity by circumventing the need for manual selection of reference points on the generated T2* curve.

Consistent with previous reports using BOLD-CMR, our study demonstrates a greater fall in T2* signal during cuffing of the control versus ischemic limbs (11). The earlier leveling off in T2* signal after cuffing in CLI patients may represent a reduced physiological reserve of muscle to ischemic stress in these patients (21). Similarly, the slower rise in T2* signal seen during reactive hyperemia in CLI patients may be due to less efficient delivery of oxygenated hemoglobin to muscle.

In patients with CLI, we measured a higher Grad and SRi in the soleus compared with the lateral and deep muscle groups. This may be a reflection of the soleus’ larger muscle mass, allowing a more accurate quantification of T2* signal intensity with generation of smoother curves. The soleus muscle is also different in that it has a dual supply from both the posterior tibial and peroneal vessels and is predominantly red muscle composed of Type 1 oxidative fibers, which may also influence the BOLD signal 22, 23.

ABPI is currently used for confirming PAD diagnosis and for assessing improvements in blood flow to the foot after revascularization. Unlike BOLD-CMR, ABPI cannot, however, be measured in all patients. The present study’s results reflect this, as we were unable to measure ABPI in ∼40% of the patients with CLI. There was no correlation between ABPI values and corresponding Grad or SRi, the former measuring global blood flow into the limb rather than perfusion at tissue level. It should be noted, however, that the combination of clinical examination and ABPI remains a practical, simple, and useful tool to initially assess of the critically ischemic limb.

Our study suggests that BOLD-CMR may help to assess changes in perfusion in the immediate post-intervention period. It can also detect incremental changes in perfusion related to the status of the inflow vessels (i.e., patency of the SFA post-revascularization in the present study), which ABPI does not discriminate. All of the patients in the intervention group resolved symptoms, and therefore, future studies should include patients with a high risk of intervention failure to see if, at an early stage, BOLD-CMR can reliably identify those who require further intervention. BOLD-CMR sequences could also readily be added to MR angiography studies to provide both functional and anatomic information simultaneously.

### Study limitations

One limitation of BOLD-CMR is its utility in patients undergoing distal bypass, where edema and hematoma in the field of view may preclude accurate assessment of T2* signal. Occlusion of the bypass with prolonged cuffing also may threaten the graft. Not all CLI patients were able to remain still for the duration of the BOLD-CMR due to the presence of rest pain when supine. Although dynamic imaging took only 12 min, this remains still sufficiently long for some patients not to tolerate the scan.

## Conclusions

The present study shows that BOLD-CMR consistently detects perfusion deficits in the ischemic limb and quantifies improvements in perfusion after revascularization. A reliable method for assessing segmental muscle perfusion in the limb would facilitate rapid detection of changes in perfusion to determine adequacy of revascularization procedures. BOLD-based perfusion metrics may also provide assessment of pharmacological and cell-based angiogenic therapies being tested as part of clinical studies and merits further investigation in a clinical trial.

Perspectives **COMPETENCY IN PATIENT CARE AND PROCEDURAL SKILLS:** Methods to accurately measure tissue perfusion in the ischemic limb are relatively cumbersome and currently not widely adopted in clinical practice, but CMR imaging techniques are being developed to address this. BOLD and ASL CMR can measure perfusion in the calf musculature and detect changes in perfusion after limb revascularization.

**TRANSLATIONAL OUTLOOK:** Clinical studies are needed in which CMR perfusion measurements are combined with CMR angiographic imaging to guide management and outcomes are compared to those resulting from conventional assessment strategies.
