# Supplementary material for: Blood Oxygenation Level-Dependent CMR-Derived Measures in Critical Limb Ischemia and Changes With Revascularization
Source: J Am Coll Cardiol. 2016 Feb 2;67(4):420–31. doi: 10.1016/j.jacc.2015.10.085 (PMC4728170; doi:10.1016/j.jacc.2015.10.085)
Supplement: Online Data [file mmc1.docx]

**ONLINE APPENDIX**

**Supplemental Methods**

**1. Limb cuffing**

Reactive hyperemia by arterial occlusion was used to elicit T2* changes. A 20 cm wide inflatable cuff (Topspins Inc., Ann Arbor, Michigan USA) was placed around each thigh and both cuffs manually inflated simultaneously over 20 seconds to 50 mm Hg above systolic pressure, kept inflated for 5minutes (ischemic phase), and simultaneously deflated over 5 seconds (reactive hyperemia). The same investigator carried out all cuff inflations/deflations. Continuous dynamic scanning began with 2 minutes of baseline imaging prior to cuff inflation and continued for 5 minutes after cuff deflation.

**2. Automated Analysis of T2* Signal Curve Parameters**

The Gradient (Grad) was calculated by taking the first derivative of each point after cuff deflation until the maximum T2* value, and then taking the mean for the highest 10 values during this period. Signal reduction during the ischemic phase (SRi) was calculated as a percentage drop in T2* signal from mean during initial baseline to the minimum T2* at the end of the ischemic phase. Baseline at the start was calculated as a mean of T2* signal over 90 seconds prior to cuffing, excluding 15seconds at the start of the scan and 15seconds before cuff was inflated. Baseline at the end was measured as a mean of the last 90seconds of dynamic imaging with BOLD-CMR. Minimum T2* value was calculated as the lowest T2* signal intensity prior to reactive hyperemia and similarly maximum T2* value was calculated as the highest signal after cuff deflation and initiation of reactive hyperemia. Time to peak (TTP) was measured as time from cuff deflation till the maximum T2* in reactive hyperemia. The time to half ischemia (THIM) was calculated as the time from the start of the scan until 50% reduction in T2* signal after cuff inflation.

**3. Histological assessment of vascularity in the limb**

The five separate muscle groups analyzed with BOLD-CMR (anterior, lateral, soleus, gastrocnemius, deep posterior) were biopsied at the imaging slice level (Supplementary Figure 6A). From the proximal section, muscle biopsies were taken from the same five muscle groups in below knee amputations and three biopsies taken from 3 muscle groups in above knee amputations (anterior, posterior, medial). Tissue biopsies were fixed in 4% paraformaldehyde for 30 minutes and dehydrated in PBS/15% sucrose for 12 hours, PBS/30% sucrose for 48 hours and PBS/40% sucrose for a further 24 hours. Samples were dried, embedded in optimal cutting temperature compound (OCT, Sakura Finetek Inc., Torrance, California) and frozen in isopentane pre-cooled in liquid nitrogen. Immunohistochemical staining was carried out using 7μm-thick frozen sections with the fluorescently labelled antibodies: endothelial cell marker (CD31, Alexa Fluor 488-conjugated, 0.6mg/ml, Novus Biological, Abingdon, UK) and basement membrane marker (laminin, Dylight 488-conjugated with Cy3 secondary, 0.85mg/ml, Novus Biological, UK). The capillary:fiber (C:F) ratio was determined by analysis of 4 fields of view in 2 sections of each biopsy obtained 5 mm apart. Comparison of C:F ratio was made between muscle biopsies proximal to the amputation site and those taken at the level of CMR. For each patient the C:F ratio from poorly perfused muscle at the level of BOLD-CMR imaging was normalized with the C:F ratio from proximal well perfused muscle and correlated with Grad and SRi.

**Supplemental Results**

**1. Automated Analysis of T2* Signal Curve Parameters**

Previous studies have identified TTP, THIM and minimum T2* as the parameters most consistent in discriminating between patient and control limbs. We found that 3 parameters were significantly different, using automated analysis, between patients ischemic limb and patient contralateral limbs, Grad, SRi and minimum T2* (Main Figure 4A). Although Grad is analogous to TTP, in that it measures the rate of increase during reactive hyperemia, not all CLI patients have a readily identifiable peak after reactive hyperemia, which explains why TTP was not significantly different between contralateral and ischemic limbs. Similarly, SRi measures are analogous to minimum T2* and THIM where the fall in signal during ischemia is assessed. SRi is more objective than THIM as it does not require manual selection of half point of ischemia. The reason we chose to proceed with SRi over minimum T2* is that it overcomes the problem of normalizing T2* allowing for more meaningful comparison between subjects.

**2. Histological assessment of vascularity in the limb musculature**

In the three patients who had amputations (one above knee and two below knee), both Grad (0.15±0.10ms/s) and SRi (7.74±1.05%) fell outside the range for age-matched control limbs (Grad 0.38±0.17ms/s and SRi 13.77±6.33%, Supplementary Figure 6B), confirming impaired perfusion in the ischemic limb at the level of BOLD-CMR imaging. Muscle biopsies taken from the same level at the time of amputation had a significantly lower capillary:fiber (C:F) ratio compared with those taken from the well-perfused musculature proximal to the amputation level (2.97±0.79 vs. 4.15±0.92, p<0.001, Supplementary Figure 6C). The C:F ratio in muscle biopsies taken at calf level correlated with Grad (r=0.64, p<0.01) but not SRi (0.25, p>0.05)(Main Figure 6)

**Online Figure 1**

**Online Figure 1. Differences in ABPI between the different study groups.** Significant differences are seen in ABPI measured in age-matched controls, patient contralateral limbs and patient ischemic limbs (one-way ANOVA).

**Online Figure 2**

**Online Figure 2. T2* signal curve reproducibility.**

**A. Inter-user Reproducibility:** Example curve from the soleus muscle of the same subject analyzed by 2 independent users showing excellent reproducibility with automated analysis. No significant difference is seen for Grad and SRi between users when analyzing BOLD-CMR (paired t test).

**B. Inter-scan Reproducibility:** The same user analyzed repeat BOLD-CMR in the same subject and example curve shown for the soleus muscle showing good reproducibility interval scans. Analysis of curve parameters shows no significant difference for Grad and SRi between interval scans (paired t-test).

**Online Figure 3**

**Online Figure 3. Correlation of ABPI with corresponding BOLD-CMR parameters.** No correlation was found between ABPI measurements and corresponding **(A)** Grad and **(B)** SRi values in the critically ischemic limb

**Online Figure 4**

**_____________________________________________________________________**

**Online Figure 4. Comparison of BOLD-CMR in CLI patients based on age, smoking and diabetes. (A)** Patients <65 years (n=13) have a significantly higher Grad and SRi compared with patients over 65 years (n=21). **(B)** No significant difference was seen between smokers (n=26) and non-smokers (n=8); and **(C)** No significant difference was found between diabetic (n=13) and non-diabetic patients (n=21)(all unpaired t-test).

**Online Figure 5**

**Online Figure 5. Correlation of ABPI and BOLD-CMR parameter changes after revascularization.** The fold change in ABPI after limb revascularization did not correlate with either **(A)** Grad or **(B)** SRi.

**Online Figure 6**

**Online Figure 6. Capillary:fiber Ratio Analysis. (A)** Muscle biopsies were taken from poorly-perfused muscle at the level of BOLD-CMR imaging and from normally perfused muscle proximal to the amputation level. Muscle was stained with CD31 (green) and laminin (red) and analyzed for capillary:fiber ratio. **(B)** The Grad and SRi was significantly lower in the ischemic legs prior to amputation compared with age-matched control limbs, confirming that the muscle biopsied at the level of the axial image was ischemic according to BOLD-CMR criteria (p<0.0005 for both, unpaired t-test). **(C)** A significantly higher capillary:fiber ratio was shown in the proximal better perfused muscle compared with the distal, poorly perfused muscle at the level of BOLD-CMR imaging (p<0.001 unpaired t-test).

**Online Table 1. CLI patients recruited for imaging pre and post intervention.**

|  | **Sex** | **Age** | **Rutherford** | **Disease** | **ABPI Pre** | **ABPI Post** | **Procedure** | **Crural Vessel Patent** | **Outcome** | **Follow up** |
| --- | --- | --- | --- | --- | --- | --- | --- | --- | --- | --- |
| 1 | M | 65 | 5 | SFA occlusion | +++ | +++ | SFA angioplasty and stenting | PTA/PeRA | Tissue loss healed | 17 months |
| 2 | M | 52 | 4 | CIA/EIA/POP occlusion | Low | 0.62 | Femoral endarterectomy & Iliac stenting | PTA/PeRA | Rest pain resolved | 13 months |
| 3 | M | 65 | 4 | CIA/EIA occlusion | Low | 1.06 | Femoral-Femoral crossover | 3 vessels patent | Rest pain resolved | 13 months |
| 4 | M | 69 | 4 | Aorto-Iliac/SFA occlusion | Low | 0.46 | Axillo-Femoral bypass | PTA | Rest pain resolved | 12 months |
| 5 | M | 79 | 5 | CIA/EIA occlusion | +++ | +++ | Iliac angioplasty and Stent | PTA | Rest pain resolved | 2 months |
| 6 | M | 72 | 4 | CIA/EIA occlusion | 0.51 | 0.93 | Femoral-Femoral crossover | 3 vessels patent | Rest pain resolved | 4 months |
| 7 | M | 65 | 4 | EIA stenosis, CFA occlusion | 0.22 | 0.7 | Femoral endarterectomy & Iliac angioplasty & Stenting | 3 vessel patent | Rest pain resolved | 6 months |
| 8 | M | 79 | 4 | EIA/CFA/SFA occlusion | +++ | +++ | Femoral endarterectomy & Iliac angioplasty & Stenting | 3 vessels patent | Rest pain resolved | 6 months |
| 9 | M | 62 | 4 | CIA/EIA occlusion | +++ | +++ | Iliac angioplasty and Stenting | 3 vessels patent | Rest pain resolved | 12 months |
| 10 | F | 64 | 4 | CIA/SFA occluded | 0.43 | 0.81 | Femoral-Femoral crossover | 3 vessels patent | Rest pain resolved | 19 months |
| 11 | F | 69 | 4 | SFA occlusion | +++ | +++ | SFA angioplasty and stenting | 3 vessels patent | Rest pain resolved | 1 month |
| 12 | M | 75 | 5 | EIA stenosis, SFA occlusion | 0.58 | 0.95 | Iliac stenting & SFA angioplasty | PeRA | In stent stenosis angioplasty, Improved | 16 months |
| 13* | M | 59 | 5 | EIA/SFA occlusion | 0.38 | 0.53 | Femoral endarterectomy & Iliac/SFA angioplasty & stenting | ATA/PeRA | Stent occlusion needing bypass | 17 months |

**Online Table 1. CLI patients recruited for imaging pre and post intervention.** Table shows the CLI patients recruited for BOLD MRI before and after intervention along with extent of disease and outcome. *Patient excluded from analysis. (+++ = noncompressible arteries, CIA = common iliac artery, EIA = external iliac artery, CFA = common femoral artery, SFA = superficial femoral artery, ATA = anterior tibial artery, PTA = posterior tibial artery, PeRA = peroneal artery, Low, inaudible Doppler signal).
